# Rapid, Point-of-Care Microwave Lysis and Electrochemical Detection of *Clostridioides difficile* Directly from Stool Samples

**DOI:** 10.3390/bioengineering11060632

**Published:** 2024-06-20

**Authors:** Lovleen Tina Joshi, Emmanuel Brousseau, Trefor Morris, Jonathan Lees, Adrian Porch, Les Baillie

**Affiliations:** 1 Faculty of Health, University of Plymouth, Plymouth PL4 8AA, UK; 2 School of Engineering, Cardiff University, Cardiff CF24 3AA, UK; brousseaue@cardiff.ac.uk (E.B.); leesj2@cardiff.ac.uk (J.L.); porcha@cardiff.ac.uk (A.P.); 3 Anaerobe Reference Laboratory, Public Health Wales, University Hospital of Wales, Cardiff CF14 4XW, UK; trefor.morris@wales.nhs.uk; 4 School of Pharmacy & Pharmaceutical Sciences, Cardiff University, Cardiff CF10 3NB, UK; bailliel@cardiff.ac.uk

**Keywords:** microwaves, lysis, DNA detection, *Clostridioides difficile*, spores, electrochemistry, bioengineering, point-of-care, biosensors

## Abstract

The rapid detection of the spore form of *Clostridioides difficile* has remained a challenge for clinicians. To address this, we have developed a novel, precise, microwave-enhanced approach for near-spontaneous release of DNA from *C. difficile* spores via a bespoke microwave lysis platform. *C. difficile* spores were microwave-irradiated for 5 s in a pulsed microwave electric field at 2.45 GHz to lyse the spore and bacteria in each sample, which was then added to a screen-printed electrode and electrochemical DNA biosensor assay system to identify presence of the pathogen’s two toxin genes. The microwave lysis method released both single-stranded and double-stranded genome DNA from the bacterium at quantifiable concentrations between 0.02 μg/mL to 250 μg/mL allowing for subsequent downstream detection in the biosensor. The electrochemical bench-top system comprises of oligonucleotide probes specific to conserved regions within *tcdA* and *tcdB* toxin genes of *C. difficile* and was able to detect 800 spores of *C. difficile* within 300 µL of unprocessed human stool samples in under 10 min. These results demonstrate the feasibility of using a solid-state power generated, pulsed microwave electric field to lyse and release DNA from human stool infected with *C. difficile* spores. This rapid microwave lysis method enhanced the rapidity of subsequent electrochemical detection in the development of a rapid point-of-care biosensor platform for *C. difficile*.

## 1. Introduction

*Clostridioides difficile* is an anaerobic spore-forming pathogen implicated as the primary cause of antibiotic-associated diarrhea, and healthcare-acquired infections (HCAIs), globally [1]. Its spores are implicated in long-term survival, biocide, and heat resistance resulting in transmission of the pathogen [2,3]. *C. difficile* Infection (CDI) causes ~29,000 deaths per year in the USA and 8382 deaths per year in Europe, with current data showing an increased incidence of CDI after the COVID-19 pandemic [4,5].

Patients usually acquire CDI when spores are transmitted via the fecal to oral route in healthcare environments, either through direct or indirect contact with contaminated areas or an infected patient [6]. Once spores have been ingested, they germinate into vegetative bacteria in response to bile salts present in the colon, and toxigenic strains secrete two clostridial toxins, Toxin A (TcdA) and Toxin B (TcdB), and a Binary toxin [7]. The production of these toxins can contribute to patient symptoms ranging from diarrhea to pseudomembranous colitis and toxin megacolon [8].

Diagnostic laboratories regularly employ algorithms to detect toxigenic *C. difficile* in symptomatic hospitalized patients. This usually involves rapid immunogenic screening for the presence of the glutamate dehydrogenase antigen (GDH) on *C. difficile* vegetative bacteria, in conjunction with an enzyme immunoassay (EIA) to detect the presence of TcdA and TcdB [9,10]. These algorithms have yet to be standardized globally; therefore, the performances of differing diagnostic test algorithms are directly compared to the gold standard cell culture neutralization assay (CCTA) in studies, often generating conflicting results and high operation costs [11,12]. While these algorithms improve patient diagnosis, they also increase the time taken to detect the pathogen, meaning results are often not available for hours. After sampling, the specimen must be transported to and tested in the laboratory. Therefore, results from potentially toxigenic samples may be compromised due to the degradation of toxins within the stool sample, affecting the reliability of results [13]. This, coupled with increased evidence of antimicrobial resistance in clinical isolates of the pathogen, highlights the importance of rapidly diagnosing CDI patients to reduce pathogen transmission, and deliver rapid antibiotic therapy. Therefore, a point-of-care (PoC) diagnostic test with a rapid (in under 10 min) result would increase the speed of patient diagnosis and assist in the implementation of infection control procedures.

To address this unmet need, we are developing a simple PoC test capable of operation with minimal training at the patient’s bedside or within a doctor’s appointment. This aims to support real-time clinical diagnosis of patients with suspected CDI prior to administration of an antibiotic, hence assisting appropriate antibiotic stewardship and prescribing [14]. We have designed a compact lysis platform that uses bespoke targeted microwave irradiation to lyse *C. difficile* spores and bacteria to release DNA which is then detected within an electrochemical biosensor assay platform. Our previous 2014 study [7] used a conventional kitchen microwave oven (operating at 2.45 GHz) to release DNA using a gold “bow tie” lysis slide, with a microwave-accelerated metal-enhanced fluorescence (MAMEF) assay technology for subsequent detection of DNA from *C. difficile* bacteria and spores, which was operated using large table-top laser platforms [7]. While the MAMEF and gold tie microwave method demonstrated high sensitivity and DNA release, neither was suitable for miniaturization or portable diagnostic PoC applications. The bespoke microwave system used in this study leverages solid-state power generation, with pulsed capability and full control over the microwave electric field to support bacterial cell and spore lysis [15].

Microwaves are a type of electromagnetic radiation with free-space wavelengths ranging from 1 m to 1 mm, with the frequency ranging between 300 MHz and 300 GHz, respectively. The microwave frequency employed in this study is centered around 2.45 GHz, which lies within the Industrial Scientific and Medical (ISM) radio band, which is reserved for such purposes [16,17]. Electromagnetic fields at a frequency of 2.45 GHz penetrate aqueous samples up to a few cm deep, and so enable uniform volumetric heating [15,18] in a targeted and highly efficient manner. This is especially so when compared with traditional conductive heating methods, whereby the resulting heating rate is highly dependent on the thermal resistance imposed by the nature of the boundaries between the material components [19].

The use of microwaves in biomedical applications has become more common in recent years, a relevant example being the microwave-accelerated metal-enhanced fluorescence (MAMEF) detection method for DNA [7,20]. The underlying principle of the MAMEF technology is the selective heating of the water molecules via microwave power, while the metallic surface is not heated. This generates a temperature gradient between the cold metal and the warm aqueous surface, facilitating mass transport of DNA to the surface where it is recognized [7,17,20,21]. Microwaves have also been used in a Polymerase Chain Reaction (PCR) microfluidics based-system, where researchers have used a tuned microwave cavity to heat and cool DNA (as usually performed by a thermocycler) to amplify DNA [22].

**Figure 1:**
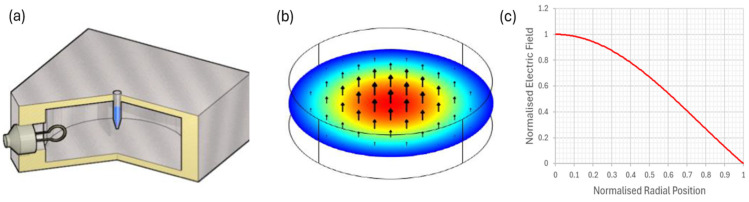
(**a**) A cylindrical aluminum cavity operating in its TM010 mode, designed to deliver 2.45 GHz of precise microwave radiation to the bacterial sample. An adjustable coupling loop is used to match the cavity to the microwave source to ensure maximum power delivery to the sample. (**b**) The normalized electric field distribution in the TM010 mode; the sample tube is placed in the region of the high microwave electric field, near the axis of the cavity, with the field parallel to the axis of the tube. (**c**) The well-known Bessel function form J0 (2.405x) for the radial dependence of electric field magnitude.

This study describes the use of a bespoke, single-mode microwave-resonant cavity with solid-state electronics to deliver constant (100% duty cycle) and pulsed microwaves at a range of duty cycles to the sample (Figure 1) [15]. The cavity allows for targeted and directed microwaves at a peak absorbed power of 30 W rms milli-Watts to penetrate the sample, which in this instance is used to break open bacteria and spores of *C. difficile* to release DNA within 5 s [15]. The electrochemical platform uses previously designed [7] oligonucleotide DNA probes specific to the *tcdA* and *tcdB* genes of toxigenic *C. difficile* to detect its presence within human stool samples. Initially, the microwaved target DNA is captured by an anchor DNA probe linked to biotin that is attached to the surface of the sensor, which is impregnated with streptavidin, via a biotin/streptavidin link. A second reporter DNA probe, which has horse radish peroxidase (HRP) added, generates an electrochemical potential when bound to the three-piece DNA complex, producing a measurable voltametric signal. Herein we describe the rapid detection of *C. difficile* within stool from infected patients within 10 min, using the combined approach of microwave lysis and electrochemical detection. This study examines the utility of using microwave power to release DNA for subsequent detection within an electrochemical DNA biosensor platform in the development of a PoC device.

## 2. Materials and Methods

### 2.1. Bacterial Strains and Growth Conditions

The clinical isolates of *C. difficile* used in this study were toxigenic clinical isolate DS1813 PCR Ribotype (RT) 027 (B1NAP1/027) containing *tcdA* and *tcdB* genes within its genome, and a non-toxigenic DS1684 PCR RT 010 with no toxin genes (or pathogenicity locus) within its genome. Strains were obtained from the National Anaerobic Reference Unit (Cardiff, Wales, UK). Unless otherwise stated, all organisms were stored as spores at 4 °C. Brain heart infusion (BHI) agar and broth (Oxoid Ltd., Basingstoke, UK) supplemented with 0.1% sodium taurocholate was used as a culture medium. The anaerobic incubation methods used were as previously published [3]. Clinical fecal samples submitted to Public Health Wales for diagnostic analysis were cultured on non-selective Fastidious Anaerobic Agar (FAA) and were residual, anonymous, discarded diagnostic material. These did not require ethical approval or consent in the United Kingdom.

### 2.2. Genomic DNA Extraction from C. difficile Using Chelex 100*^®^*

Genomic DNA (gDNA) was extracted from *C. difficile* as described previously [23]. Briefly, a single colony of *C. difficile* was harvested from a 24-h anaerobic culture on an FAA plate, and suspended in 5% (*w*/*v*) solution of Chelex-100 (Bio-Rad, Hemel Hempstead, UK). The solution was boiled for 12 min and cellular debris subsequently removed after centrifugation at 15,000× *g* for 10 min. The supernatant contained the gDNA.

### 2.3. Microwave Apparatus and Exposure Details

The microwave cavity used for the electric field exposure of *C. difficile* was of identical geometry to that reported elsewhere for the electromagnetic characterization of magnetite [24] and nano-diamond samples [25]. Briefly, the cylindrical cavity was machined from aluminum and had an internal diameter of 92 mm and an internal length of 40 mm, designed to have an unperturbed resonant frequency of the TM_010_ mode of 2.50 GHz (reduced to 2.45 GHz on dielectric loading by the sample). These dimensions ensured spectral separation of the TM010 mode from competing modes such as TE_111_ (at 4.2 GHz) and maintained a high quality (Q) factor of 8000 when the cavity was empty, both of which ensured maximum transfer of available microwave power. A bacterial sample within a 200 µL Eppendorf tube (filled with an aqueous sample occupying a volume of 170 µL) was found to reduce the Q factor to 200. Since the empty plastic Eppendorf tubes were measured in separate cavity experiments to have negligible microwave loss, ~98% of the microwave power delivered to the cavity was dissipated in the sample [18]. Microwaves were inductively coupled to the microwave magnetic field around the perimeter of the cavity via an adjustable coupling loop, made from a short-circuited N connector. This could be both rotated and moved in and out of the cavity to ensure fine control of the impedance matching at resonance. Coupling was adjusted to give a power reflection coefficient at a resonance of <−20 dB, so that at least 99% of the input power was absorbed by the cavity and its sample (about 98% of this absorbed by the sample).

**Figure 2:**
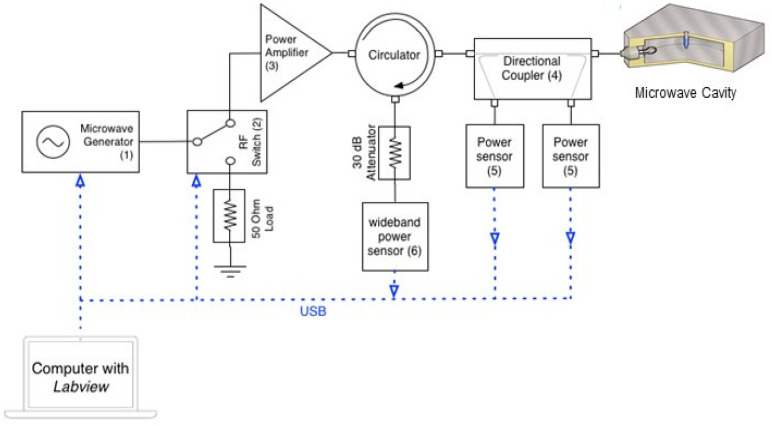
Microwave circuitry. The cavity is excited by a highly adaptable, solid-state microwave power delivery system (up to 30 W) comprising a low power source and high-power amplifier. The power sensors [5] are used to measure the incident and reflected power to ensure that maximum power transfer conditions can be attained. An additional, a wideband power sensor [6] allows the measurement of any reflected power for low duty cycles, during short pulses. The RF switch allows the microwaves to be pulsed at duty cycles ranging from 0.3% to 100% (Table S1).

A schematic of the microwave circuitry is shown in Figure 2. The solid-state microwave source (1—(Telemakus TEG27006, Telemakus, LLC, Folsom, CA, USA) provided a single-frequency microwave output at a power of 0 dBm (i.e., 1 mW rms). The RF switch (2—(Telemakus TES6000-30, Telemakus, LLC, Folsom, CA, USA) allowed the microwaves to be pulsed at duty cycles ranging from 0.3% to 100% (here we define a duty cycle to be the % ratio of the time the microwave power is on to the time it is off, as a percentage of the switched waveform cycle). The microwave power amplifier (3—(Mini-circuits ZHL-30W-262, Mini-Circuits, Brooklyn, NY, USA) had a maximum output power of approximately 30 Watts and a gain of approximately 50 dB over the system bandwidth of 2.0 to 2.5 GHz. The combination of the directional coupler (4—(Mini-circuits ZABDC20-322H, Mini-Circuits, Brooklyn, NY, USA) and the two precision power sensors (5—(Telemakus TED6000-50, Telemakus, LLC, Folsom, CA, USA) allowed simultaneous measurements of both the transmitted and reflected microwave powers of incident on and reflected from the sample-loaded cavity, respectively. The wideband power sensor (6—(Rhode & Schwarz NRP-Z81, Rohde & Schwarz USA, Inc., Columbia, MD, USA) had a maximum video bandwidth of 30 MHz and was capable of measuring pulses as small as 50 ns. The sensor was triggered to allow accurate measurement of the reflected power from the microwave pulses applied to the cavity and used to confirm that any change in reflection coefficient during the pulse cycle, due to sample heating, was minimal. All equipment was controlled by National Instruments LabVIEW 2015 software, which provided a user interface and also recorded the power readings from all of the power sensors. The maximum delivered power of 30 W rms gave a maximum local microwave electric field amplitude of approximately 15 kV/m within the sample. For continuous microwave exposure (i.e., at 100% duty cycle), this high electric field gives rise to an initial sample heating rate of over 40 °C/s and in practice, samples were found to boil in around 4 s. To ensure a high electric field yet negligible global sample heating, a low duty cycle of only a few percent was used in practice.

In this study we utilized continuous microwaves (100% duty cycle), and pulsed microwaves at duty cycles of 1%, 10%, and 100% to examine DNA release from *C. difficile* spores suspended in varying matrices of sterile water and human feces.

The subject of the microwave dosage is a very important one. In Figure 1 we show the theoretical electric field associated with the TM010 mode of the cylindrical cavity, normalized so that it takes the dimensionless value of 1 on-axis. This is the usual Bessel function dependence J_0 (x) and we placed samples on-axis, parallel to the axis, to maximize the effectiveness of the electric field and to minimize depolarization effects associated with the long, thin Eppendorf tubes. However, this was not the electric field within the sample to which the spores were exposed, which we estimate in magnitude below.

We did not want to boil the sample as this would have denatured the target DNA. At full 30 W continuous microwave power (CW) we estimated a heating rate of 40 °C/s for a sample of 170 µL of deionized water. This was measured by monitoring the reflected power from the cavity. As soon as the sample started to boil the bubbles of steam suddenly changed their effective permittivity and also the input match to the cavity, which introduced sudden and chaotic changes in the reflected power. Boiling occurred after 2.0 s for 30 W CW input power, giving the quoted heating rate based on a laboratory temperature of 20 °C. The sample volume of 170 µL corresponded to a heat capacity of approximately 0.7 J/°C and the dissipated power was then calculated to be approximately 28 W. This was consistent with the cavity and its aqueous sample load being impedance matched to the source, so that almost all the 30 W input power was dissipated as heat within the sample.

The most reliable way of estimating the electric field magnitude E within an aqueous sample within an Eppendorf tube (and hence representative of the absolute field to which the bacteria are exposed) is via an experiment using the power density equation, which gives the power dissipated (in W) for a sample of volume *V* of loss factor (i.e., imaginary part of the permittivity *ε*_2_, dimensionless) as

$$P=\pi f\varepsilon_{2}\varepsilon_{0}E^{2}V$$

Using this and the measured heating rate we estimate that *E* ≈ 16 kV/m, assuming the well-known loss factor *ε*_2_ ≈ 10 for water at 2.45 GHz.

Samples exposed to pulsed microwaves with low duty cycles do not boil but bacteria are still exposed to the 16 kV/m electric field when the microwaves are switched on. Even then we expected local heating, but in these instances, we measured no global increase in temperature of the sample by standard thermometry. Furthermore, an increase in sample temperature would increase the resonant frequency of the cavity and its sample in the TM010 mode, since the real part of the permittivity of water *ε*_1_ decreases with increasing temperature; we measured no such change in frequency before and after exposure to pulsed microwaves. DNA release from bacterial spores is likely to be thermally driven, but in our experiments the heat was generated on a very local scale that did not measurably increase the global sample temperature. Each bacterial spore is a very complex structure in terms of its dielectric property, and any dielectric contrast is likely to produce a non-uniform local electric field; this will result in thermal hotspots (since the local heating rate is proportional to the square of the local electric field). We do not further explore the mechanism of DNA release in this paper, other than to note that the application of pulsed microwaves is an effective and rapid means of DNA release for the spores studied here. Its origin is likely to be highly localized heating, which is immeasurable without using a microscopic temperature probe.

### 2.4. Scanning Electron Microscopy Studies of C. difficile

Spores of *C. difficile* were microwaved at a peak power of 30 W rms milli-Watts at a range of microwave duty cycles: 100%, 10%, and 1% and analyzed using Scanning Electron Microscopy (SEM) to determine if morphological changes were present. (Supplementary Information Figure S1 shows SEM results for *C. difficile* spores exposed to duty cycles ranging from 0%, 0.3%, 1%, 3% 10%, 33%, and 100%. Table S1 shows microwave duty cycle information). After microwaving, 20 µL spores of *C. difficile* strain DS1813 RT027 were inoculated onto a clean microscope slide and heat fixed [3]. Non-microwaved spores were used as a comparative control. Slides were sputter coated with metal using a gold palladium sputtering target (60% Au and 40% Pd from Testbourne Ltd., Basingstoke, UK) and argon as the sputtering gas. Images were taken on a scanning electron microscope (model XB1540 from Carl Zeiss, Jena, Germany) using an accelerating voltage of 5 kV. Forty spores per sample were viewed at magnifications of ×82,000 and ×31,000.

### 2.5. Measurement of DNA Released from Microwave Irradiated Samples

DNA quantification was performed using the Qubit 3.0 Fluorometer (Life Technologies, Renfrew, UK) as per the manufacturer’s instructions before and after microwave exposure at 1%, 10%, and 100% DC. Specifically, we were interested in quantifying single-stranded DNA (ssDNA) release to support subsequent detection in the electrochemical biosensor assay. Each measurement was repeated in triplicate and DNA yields were measured in µg/mL.

### 2.6. Electrochemical Nucleic Acid Detection of tcdA and tcdB Genes within DNA Released from Microwaved C. difficile in Water

DNA probes used to detect *tcdA* and *tcdB* genes of toxigenic *C. difficile* are as previously specified [7]. For utilization within the electrochemical DNA biosensor detection system, anchor DNA probes were labelled with biotin at the 5′ region and the reporter probes were directly labelled at the 3′ end with enzyme HRP. The anchor probe (40 µM anchor probe) was bound to the surface of the silver-ink printed acetate sensor coated with 80 µg/mL Streptavidin (Vantix, Cambridge, UK) via a biotin/streptavidin interaction. Subsequently; 50 µL target DNA lysed from *C. difficile* via microwaving was added to the anchor probe and then 40 µM reporter probe was added, forming a three-piece DNA assay complex on the biosensor [26]. The three-piece DNA assay complex (shown in Supplementary Materials Figure S2) was washed with potassium phosphate buffer (pH 7.8) to remove any unbound DNA, and an enzyme substrate was added to generate voltage proportional to the number of copies of the target gene within the sample. The electrochemical signal was generated by horseradish peroxidase (HRP), catalyzing the electro-reduction of hydrogen peroxide in the presence of a hydrogen donor, in this case o-phenylenediamine (OPD), resulting in the transfer of an electron from the sensor to the OPD substrate. Toxigenic strain DS1813 was used as the test strain, and non-toxigenic DS1684 was used as the negative control strain.

### 2.7. Electrochemical Nucleic Acid Detection of tcdA and tcdB Genes from Clinical Fecal Specimens

A panel of 50 blinded clinical fecal specimens submitted to Public Health Wales for diagnostic analysis (University Hospital Wales, Cardiff, UK) were tested for the presence of *tcdA* and *tcdB* genes. The blinded samples were previously tested for the presence of glutamate dehydrogenase and toxin A and toxin B using an Enzyme Immunoassay (EIA) with a limit of detection (LoD) of >0.8 ng/mL Toxin A and >2.5 ng/mL for Toxin B (Techlab, London, UK) [27] at the University Hospital of Wales’ Public Health laboratories; of these, 10 samples were *C. difficile* negative and the remaining 40 were *C. difficile* positive. For the toxin assay, 50 µL of liquid stool sample was diluted to a volume of 200 µL. Public Health Wales also undertook selective agar culture to check each fecal sample for the presence of *C. difficile.*

To detect the presence of the pathogen in clinical samples using our microwave-enhanced method, 900 µL of loose stool was diluted with 100 µL Phosphate Buffered Saline (PBS) to enhance viscosity and was vortex mixed for 2 min. Then, 170 µL of that sample was microwaved at a peak power of 30 W rms milli-Watts at a duty cycle of 10% DC to release target DNA. Subsequently, 50 µL of the microwaved sample was added to the electrochemical reporter platform for detection purposes. Prior to, and post microwave exposure, the colony-forming counts (cfu) of *C. difficile* were enumerated to determine whether there was a reduction in recoverable *C. difficile* after microwave exposure [3]. Samples were diluted in Fastidious Anaerobic Broth (EO labs, York, UK) and enumeration was performed on Braziers CCEY Agar and incubated for 48 h under anaerobic conditions, as described previously [3].

### 2.8. Statistical Analysis

Data are expressed as means ± SEM. Two-sample *t*-tests and One-way ANOVA tests were performed using Minitab 19 (Minitab Inc., State College, PA, USA).

## 3. Results

### 3.1. Microwave-Mediated Spore and Vegetative Cell Lysis

**Figure 3:**
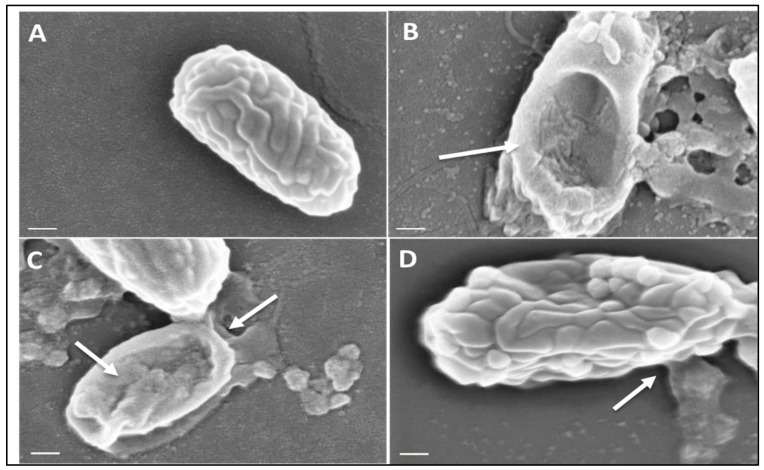
Scanning Electron Microscopy of *C. difficile* spores before and after microwaving. Spores of strain DS1813 were imaged under SEM before and after microwaving at a peak power of 30 W rms milli-Watts at 100%, 10%, and 1% duty cycles (DC) for 5 s. A total of 40 spores per sample were imaged per DC at ×82,000 magnification, with the spores chosen here representative of spores consistently seen within the sample. White arrows indicate areas of lysis and morphological damage. (**A**) A control spore of DS1813 which was not exposed to microwaves. (**B**) DS1813 exposed to constant microwaves at 100% DC. Damage to the spore structure and debris is clearly visible on this spore. (**C**) DS1813 exposed to pulsed microwaves at 10% DC. Some damage to the spore structure is visible at its terminal end. (**D**) DS1813 exposed to pulsed microwaves at 1% DC. There is no visible damage to the spore structure. (Image scale bar = 200 nm).

While relatively small amounts of mother cell-derived DNA will adhere to the surface of the spore, the majority of target DNA is sequestered within the spore itself. Our microwave-based lysis approach has been developed to break open the spore in a controlled manner to release the internal genomic DNA and increase the sensitivity of this assay. The microwave lysis method will also release gDNA from vegetative cells of *C. difficile* which can be detected downstream in the electrochemical assay. As in Figure 3B, exposure to constant microwaving at a peak power of 30 W rms milli-Watts at 100% DC caused major disruption to the spore structure. The magnitude of this damage increased with the level of microwave exposure (Figure 3A–D and Figure S1, Table S1).

### 3.2. The Release of Target DNA from Microwaved C. difficile Spores

**Figure 4:**
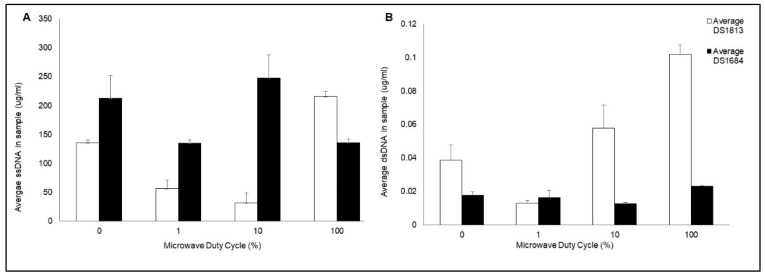
Quantification of double and single-stranded DNA released from microwaved *C. difficile* spores. Spores of toxigenic DS1813 and non-toxigenic DS1684 strains were microwaved a peak power of 30 W rms milli-Watts at a range of duty cycles (0%, 1%, 10%, 100%) for 5 s, each at a concentration of 1.67 × 10^7^ spores/mL. The single-stranded (ss) and double-stranded (dsDNA) was quantified via Qubit Fluorometer 3.0. Each test was performed in triplicate (*n* = 3) (**A**) Concentration of ssDNA in samples of DS1813 and DS1684 (ug/mL). (**B**) Concentration of dsDNA in samples of DS1813 and DS1684 in ug/mL.

In addition to characterizing the effect of microwave exposure on the physical structure of the spores, the effect of different microwave duty cycles on the release of double-stranded (ds) and single-stranded (ss) DNA was determined (Figure 4A,B). Quantification of ssDNA was particularly important as it is this form of DNA which is recognized by our pathogen-specific oligonucleotide DNA probes. Prior to microwave exposure we observed differences in the concentration of ss and ds DNA, with the single-stranded variant being the most concentrated. When the ratio of single- to double-stranded DNA was examined, the biggest relative difference was seen for DS1684 spores (ss/ds ratio 11,806) compared to DS1813 spores (3400) suggesting that DS1684 spores may carry more surface-associated extracellular DNA [26].

The quantity of ssDNA and dsDNA released from each spore type varied depending on the microwave duty cycle and level of microwave exposure. Treatment of DS1813 spores with pulsed microwave powers of 1% duty cycle (DC) and 10% DC resulted in a significant decrease in the concentration of ssDNA by 58% (Figure 2A; two-sample *t* test; *p* = 0.017) and 76% (two-sample *t* test; *p* = 0.013), respectively, when compared to spores prior to microwave exposure. In contrast, upon exposure to 100% DC, the concentration of ssDNA significantly increased by 59% (two-sample *t* test; *p* = 0.007) when also compared to control spores which were not exposed to microwaves. A one-way ANOVA established that there was a highly significant difference between the concentrations of ssDNA released when spores were treated with varying microwave exposures for DS1813 (*p* = 0.000), and DS1684 (*p* = 0.035).

The concentration of dsDNA significantly decreased by 68% following exposure to 1% DC (two-sample *t* test; *p* = 0.060), in a similar fashion to the ssDNA levels. In contrast, the concentration of dsDNA released increased by 150% as the level of microwave exposure increased at 100% DC (Figure 2B; two-sample *t* test; *p* = 0.014). One-way ANOVA determined a significant difference between the concentrations of dsDNA released when spores were treated with varying microwave exposures for DS1813 (*p* = 0.002) and for DS1684 (*p* = 0.042). Microwave treatment of DS1684 spores (30 W rms milli-Watts) showed a different pattern of ss and dsDNA release than seen for DS1813. Following exposure with 1% DC an insignificant decrease in ssDNA concentration was observed (two-sample *t* test; *p* = 0.108) which was then followed by an increase at 10% DC (two-sample *t* test; *p* = 0.461) and at 100% DC (two-sample *t* test; *p* = 0.110). However, the release of dsDNA decreased from 0% DC through to 1% DC (two-sample *t* test; *p* = 0.720) and then increased at 100% DC (two-sample *t* test; *p* = 0.057) demonstrating that DS1684 spores differ in their response to microwaves when compared to DS1813.

### 3.3. Electrochemical Detection of tcdA and tcdB in Microwaved C. difficile

The specificity of the toxin-specific DNA probes following microwave treatment was determined using the Vantix^TM^ electrochemical reporter system (Figure S2) [27]. This reporter system generates a voltage signal which is proportional to the DNA concentration in the sample. Spores of toxigenic *C. difficile* DS1813 and the non-toxigenic control DS1684 (which lacks the toxin gene targets and pathogenicity locus) at a concentration of 1.33 × 10^4^ spores/mL were exposed to 1%, 10%, and 100% DC of microwaves at a peak power of 30 W rms milli-Watts for 5 s. DNA released from spore lysis was then screened for the presence of toxin genes *tcdA* and *tcdB* using the Vantix^TM^ electrochemical reporter system.

**Figure 5:**
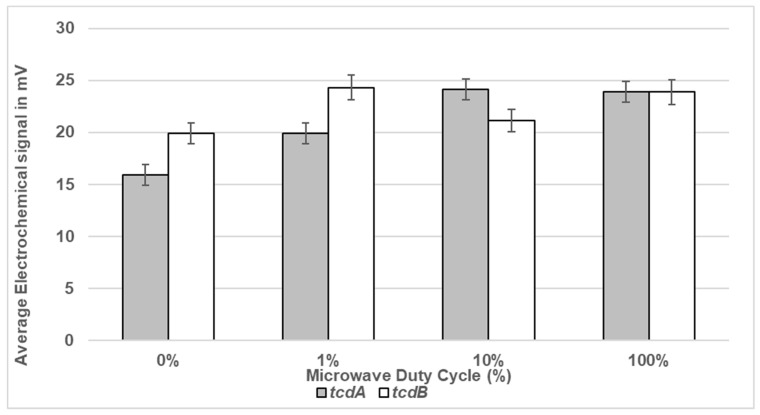
Electrochemical detection of toxigenic *C. difficile* spores suspended in sterile water following exposure to microwaves. Spores of toxigenic strain DS1813 and non-toxigenic DS1684 at a set concentration of 1.33 × 10^4^ spores/mL were exposed to duty cycles of 1%, 10%, and 100% for 5 s. Spores which were not microwaved were used at controls (0% DC). This equates to 665 spores within the 50 µL of the assay sample. The microwaved spore samples were then introduced to the Vantix^TM^ electrochemical detection system and tested for the presence of toxin genes *tcdA* and *tcdB*. The results above show voltage signals measured from toxigenic DS1813. The results from the toxin-negative DS1684 strain did not generate a measurable signal (0 mV). Each result represents the mean of two independent tests (*n* = 2).

As shown in Figure 5, exposure of DS1813 spores to 100% DC microwave power for 5 s gave the strongest signal (milliVolts) for both toxin-specific oligonucleotide probes. The signal increased as the microwave exposure increased. As expected, no measurable toxin-specific signal was observed for DS1684 spores under any of the test conditions (0 mV).

### 3.4. Determination of the Lower Limit of Electrochemical Detection in Sterile Water

**Figure 6:**
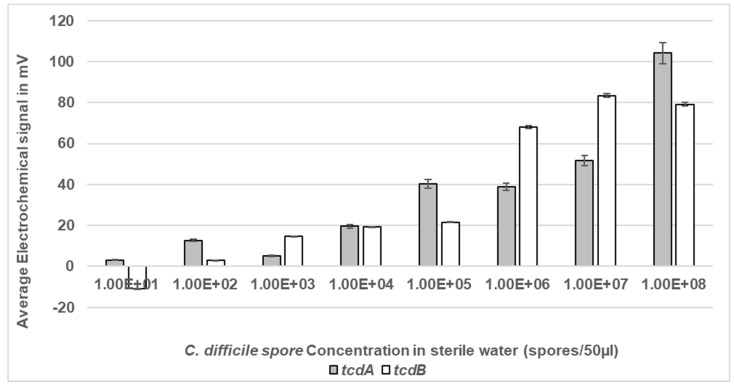
Electrochemical detection of *C. difficile* spores at a range of concentrations in sterile water. Spores of toxigenic strain DS1813 and non-toxigenic DS1684 at concentrations ranging from 1 × 10^1^ spores within a 50 µL sample to 1 × 10^8^ spores within a 50 µL sample were exposed to microwaves at 100% DC for 5 s. The microwaved spore samples were then introduced to the Vantix^TM^ electrochemical detection system and tested for the presence of both toxin genes *tcdA* and *tcdB*. No signal was detected for any of the DS1684 spore concentrations tested (0 mV). Each result represents the mean of two independent tests (*n* = 2).

The lower limit of detection (LoD) of the electrochemical detection assay was determined using a dose response. A range of spore concentrations suspended in sterile water were microwaved at a peak power of 30 W rms milli-Watts at 100% DC in sterile water and the lysed spores were then measured for LoD within the Vantix^TM^ electrochemical reporter system. The LoD for *tcdA* was 1 × 10^2^ spores/mL which equated to five spores within a 50 µL sample, whilst for *tcdB* the LoD was 1 × 10^3^ spores/mL equating to 50 spores in a 50 µL sample (Figure 6). As expected, no signal was detected at any of the DS1684 spore concentrations tested (0 mV).

### 3.5. Determination of the Lower Limit of Electrochemical Detection in Feces

**Figure 7:**
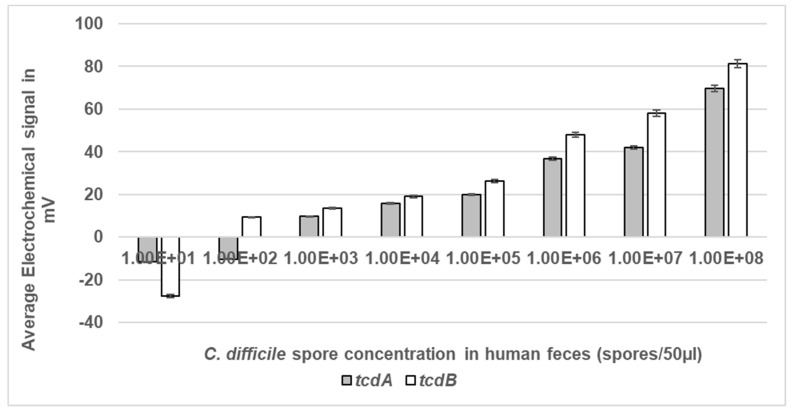
Electrochemical detection of *C. difficile* spores at a range of concentrations in human feces. Spores of toxigenic strain DS1813 and non-toxigenic DS1684 at concentrations ranging from 1 × 10^1^ spores within a 50 µL sample to 1 × 10^8^ spores within a 50 µL sample were exposed to microwaves at 100% DC for 5 s. The microwaved spore samples were then introduced to the Vantix^TM^ electrochemical detection system and tested for the presence of both toxin genes *tcdA* and *tcdB*. No signal was detected for any of the DS1684 spore concentrations tested (0 mV). Each result represents the mean of two independent tests (*n* = 2).

The ability of the system to detect *C. difficile* spores in the presence of raw, unprocessed human stool was assessed (Figure 7). Feces is the usual matrix where *C. difficile* spores are present and the gut environment contains approximately 10^12^ per g bacteria [7]. The LoD of the assay was determined using a range of spore concentrations suspended in human feces from a healthy volunteer. All samples were microwaved at a peak power of 30 W rms milli-Watts at a new DC of 33% prior to detection of liberated DNA. Then, 33% DC was used to ensure limited thermal heating of the sample and to account for the change of medium from SDW to feces. The LoD for *tcdA* was 1 × 10^3^ spores/mL which equated to 50 spores within a 50 µL sample, and for *tcdB* LoD was 1 × 10^2^ spores/mL equating to five spores in a 50 µL sample (Figure 5). The detection signals increased as the spore concentration increased. As expected, no signal was detected at any of DS1684 spore concentrations tested (0 mV).

### 3.6. Comparison of the Specificity and Sensitivity of the Microwave-Enhanced Electrochemical Detection Assay to a Toxin-Sensitive Enzyme Immunoassay (EIA)

**Figure 8:**
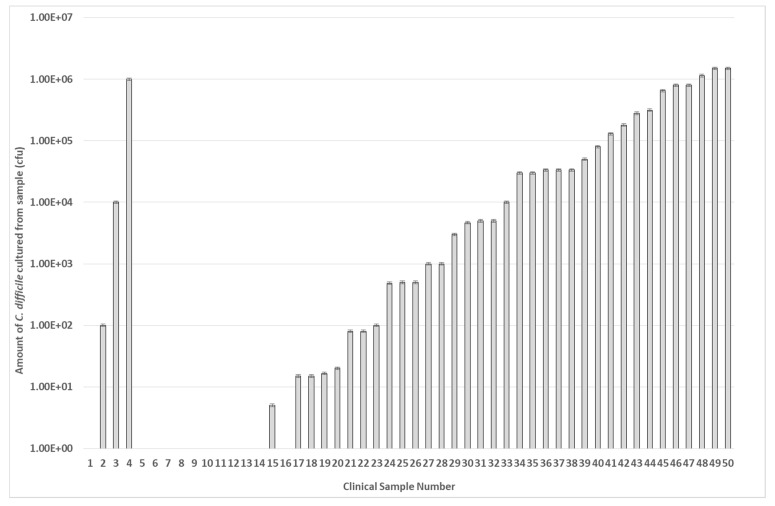
Presence of *C. difficile* in clinical stool samples. A total of 50 stool samples from patients at Public Health Wales were examined for the presence of *C. difficile* using selective agar culture. The data are arranged in order of bacterial number in each sample (cfu/mL). Samples 2, 3, & 4 are anomalous as these were deemed *C. difficile* negative by PHW. Each result represents the mean of two independent tests (*n* = 2).

The ability of the microwave-enhanced assay system to detect the presence of *C. difficile tcdA* and *tcdB* genes individually in clinical stool specimens was compared to that of the rapid Techlab *C. difficile* Tox A/B Quik Chek EIA assay [28]. The Techlab Tox A/B test is routinely used by the diagnostic service of Public Health Wales (PHW) to screen for the presence of *C. difficile*. A total of 50 discarded, anonymized human stool samples, which had been submitted to Public Heath Wales to determine the presence *C. difficile*, were cultured and screened using both rapid assays (Figure 8). Of the 50 samples examined, 16 (32%) were culture-negative for *C. difficile* via selective agar testing. Of these samples, one gave a positive result with the Techlab assay and another separate sample gave a positive result using the microwave-enhanced electrochemical assay. These differences reflect that only a 50 µL fraction of the entire stool sample was agar cultured and that it is unlikely that spores would be homogenously distributed throughout the whole stool sample during the sampling and would all germinate during the anaerobic agar culture process. When the samples were examined using the rapid assays, 32 of 34 culture-positive samples (94%) detected *C. difficile* using the microwave assay. In contrast, the EIA only detected the presence of *C. difficile* in 27 culture-positive samples (75%) (see Table 1).

## 4. Discussion

In this study we describe a microwave-enhanced bacterial lysis method combined with an electrochemical sensor platform which uses oligonucleotide DNA probes for the rapid detection of *C. difficile* toxin genes in clinical stool specimens, without the need for DNA amplification. This builds on a previous study which utilized a conventional microwave oven to liberate DNA from bacteria, with utilization of the same *C. difficile* oligonucleotide DNA probes in a microwave-accelerated metal-enhanced fluorescence (MAMEF) reporter platform [7]. Results from the MAMEF study demonstrated the specificity and sensitivity of the designed oligonucleotide probes for detection of *C. difficile* toxin genes *tcdA* and *tcdB*.

The results from this small-scale pilot study (50 samples) show disruption of *C. difficile* spores using a 2.45 GHz electric field, leading to spore lysis and the release of target DNA within 5 s (Figure 3). Extraction of DNA from clinical samples is usually time consuming and requires lysis of the bacterium or spore [29]. We have overcome this by utilizing a bespoke microwave cavity able to precisely deliver electric fields at varying intensities to the clinical sample resulting in release of ssDNA which is able to bind to our oligonucleotide capture and reporter probes and be electrochemically detected directly, without any need for purification or DNA amplification (Figure 4, Figure 5, Figure 6, Figure 7, Figure 8 and Figure S2) [7,15].

Variations in ss and ds DNA release were observed after using a range of microwave duty cycles (Figure 4A,B). This variation in lysis and overall DNA release may be attributed to the physical structure of the spores, which would influence the interactions of the microwave electric field with the spores inside the cavity [30,31,32]. It is also possible that the microwave electric field did not reach all spores within the test sample, which may be due to the natural properties of spore hydrophobicity/aggregation or changes in the generated convection current [33,34]. While we have determined that microwave irradiation does release DNA from the organism, the exact genomic mechanism of action of microwave lysis and DNA release has yet to be fully characterized and warrants further investigation.

Current Public Health England guidance for laboratory detection of *C. difficile* from clinical samples states that that a combination of two-test algorithms should be used for screening, the first of which should be a Nucleic Acid Amplification Test (NAAT) or Glutamate dehydrogenase EIA followed by a sensitive toxin-EIA test, increasing the sensitivity and accuracy of CDI diagnosis [35,36,37]. NAATs are expensive to perform and require specialist laboratory equipment to yield results with rapid approved tests such as Cepheid XpertTM still taking <1 h [38]. Current commercial NAATs include BD Gene Ohm, Cepheid Xpert, and the Cobas C diff PCR test from Roche, which only target the toxin B gene for amplification [39,40].

The ability of the microwave-based assay to detect the presence of both *C. difficile* genes *tcdA* and *tcdB* in clinical stool samples was compared to a commercially used toxin-sensitive EIA (Table 1). With this approach we have demonstrated that the microwave-enhanced assay was more sensitive in detecting culture-positive samples (94.1%) than the toxin sensitive EIA (75%) (Table 1). However, while this pilot study has shown that the microwave-enhanced assay is more sensitive than the commercial toxin-sensitive EIA, a larger clinical study is needed to determine sensitivity and specificity. A positive electrochemical detection result was obtained for sample 14 using the microwave assay when the sample was culture-negative. This could be a false positive result generated by the microwave assay as culture was unable to detect the organism [41]. The toxin-sensitive EIA was also unable to detect the presence of the toxins within the sample; correlating with the culture-negative result. However, the toxin-sensitive EIA only detects the presence of the toxins, not the genes, meaning there is a lack of sensitivity [42]; thus, there is a possibility that single copy numbers of *tcdA* and *tcdB* genes (located in the genome) may still be present within the sample through asymptomatic carriage. This possibility would need to be confirmed using a PCR test and the recommended algorithms [37].

This microwave-enhanced method detects the presence of both *tcdA* and *tcdB* genes and thus can also be used to detect asymptomatic carriage in patients—a useful screen when considering infection prevention and control of CDI. Other gold standard *C. difficile* detection methods used in the UK such as toxin-sensitive EIA currently do not provide this level of discrimination. The method we describe has distinct advantages in reducing the test time from acquiring the samples to obtaining a definitive molecular result, which is useful in triage of CDI patients in hospitals and within community settings. There is an increasing appreciation of the importance of community-acquired CDI and the role of asymptomatic carriers in transmission [36]. A study, in a setting where 42% of CDI cases were community-onset, demonstrated that testing for asymptomatic carriers plus contact precautions reduced the number of new colonization and hospital-onset CDI cases by 40%–50% and 10%–25%, respectively [43].

These results demonstrate that microwaves can be used to rapidly liberate DNA from fecal samples and that subsequent electrochemical detection (using screen-printed electrodes) may be used to screen for patients with CDI. As the majority of CDI cases occur as a consequence of prescribing broad-spectrum antibiotics to asymptomatic carriers, an indication of the presence of toxigenic *C. difficile* in a patient upon hospital admission would enable clinicians to tailor their antibiotic treatment strategy appropriately, minimizing the development of active CDI. There is potential for the methodology to be adapted and optimized for the detection of other antimicrobial resistant pathogens in a range of human sample types. Thus, microwave-extraction of DNA combined with electrochemical biosensor detection of target DNA within 10 min represents a viable, rapid, and sensitive method for the detection of toxigenic *C. difficile* at point of care.

## Figures and Tables

**Table 1:** Comparison of Signals detected from Techlab *C. difficile* Tox A/B Quik Chek test against the MW-based assay. A total of 50 stool samples from patients at Public Health Wales were examined for the presence of *C. difficile* using the routine Techlab ELISA toxin assay and the MW based detection assay. The Clinical Sample Number is the same as Figure 8 and can be directly compared. The number (1) indicates positive detection of both *C. difficile* toxin genes (*tcdA*; *tcdB*) and (0) indicates negative detection of *C. difficile* toxin genes. Each result represents the mean of two independent tests (*n* = 2).

Clinical Sample Number	Techlab Tox A/B QuikChek	MW Based Assay
0	0	0
1	0	0
2	0	0
3	0	0
4	0	0
5	0	0
6	0	0
7	0	0
8	0	0
9	0	0
10	0	0
11	0	0
12	0	0
13	0	0
14	0	1
15	0	1
16	0	0
17	1	1
18	1	1
19	1	1
20	1	1
21	1	0
22	0	0
23	1	0
24	1	0
25	0	0
26	1	0
27	1	0
28	0	0
29	0	0
30	0	0
31	1	0
32	1	0
33	1	0
34	1	0
35	1	0
36	1	0
37	1	0
38	1	0
39	1	0
40	1	0
41	1	0
42	0	0
43	1	1
44	1	1
45	1	1
46	1	1
47	1	1
48	1	1
49	1	1
50	0	1

## Data Availability

The data are available within the article.

## Supplementary Materials

The following supporting information can be downloaded at: https://www.mdpi.com/article/10.3390/bioengineering11060632/s1, Figure S1: Scanning Electron Microscopy studies. Spores were imaged under SEM before and after microwaving at a range of duty cycles between 100%–0.3%. 40 spores were imaged per DC at at magnifications of ×82,000 and ×31,000. A-C show untreated (Control) *C. difficile* spores, D-F show spores treated with 100% Duty Cycle, G-I show spores treated with 33% Duty Cycle, J-L show spores treated with 10% Duty Cycle, M-O show spores treated with 3% Duty Cycle, P-R show spores treated with 1% Duty Cycle, S-U show spores treated with 0.3% Duty Cycle. Arrows indicate areas of spore damage. Table S1: Microwave pulsed duty cycles used in this study. The varying percentage duty cycles used when microwaving *C. difficile* spores is listed. The duty cycles range from 100% (continuous microwave power) to 0.3% pulsed microwaves. The time microwave power is on and off is shown in milliseconds. Spores were microwaved for 5 s in total, which is related to the total number of pulsed microwaves (N) in the table. Figure S2: Schematic demonstrating the three piece DNA assay and the chemical detection of HRP. This DNA assay was used to detect both toxin A and toxin B detection. The anchor probe is 17 nucleotides in length and anchored to the streptavidin sensor via addition of a biotin label. The reporter probe (22 nucleotides in length) was attached to an HRP at the 3’ end. Once hybridisation and washing has occurred, the DNA sandwich complex is formed and the HRP can be detected.
