# Supplementary material for: Rapid, Point-of-Care Microwave Lysis and Electrochemical Detection of Clostridioides difficile Directly from Stool Samples
Source: Bioengineering (Basel). 2024 Jun 20;11(6):632. doi: 10.3390/bioengineering11060632 (PMC11200505; doi:10.3390/bioengineering11060632)
Supplement: Supplementary file 1 [file bioengineering-11-00632-s001.zip › bioengineering-2984878-supplementary.pdf]

bioengineering

Supplemental Material

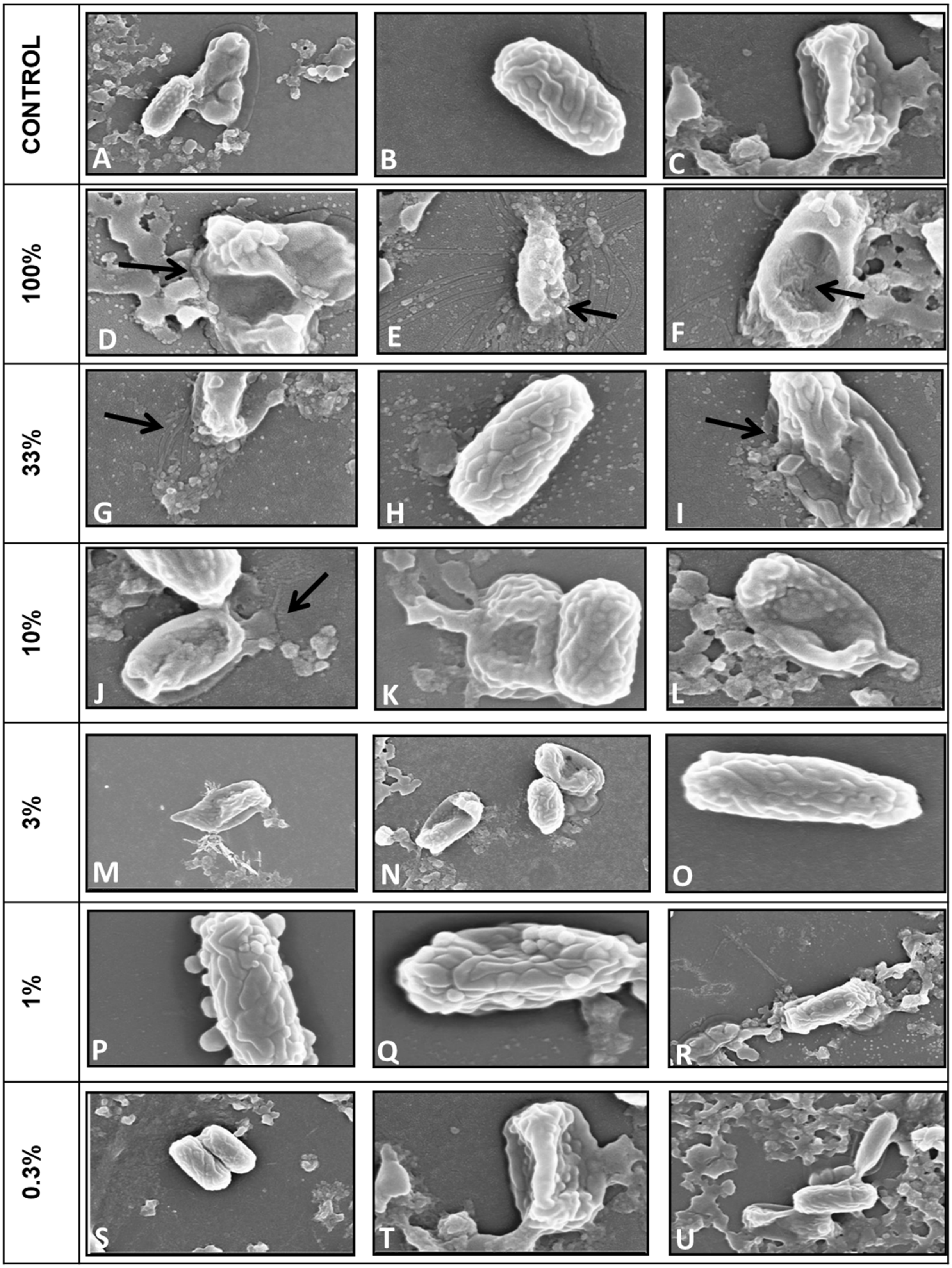

Figure S1. Scanning Electron Microscopy studies. Spores were imaged under SEM before and after microwaving at a range of duty cycles between 100%- 0.3%. 40 spores were imaged per DC at at magnifications of x82,000 and x31,000. A-C show untreated (Control) *C. difficile* spores, D-F show spores treated with 100% Duty Cycle, G-I show spores treated with 33% Duty Cycle, J-L show spores treated with 10% Duty Cycle, M-O show spores treated with 3% Duty Cycle, P-R show spores treated with 1% Duty Cycle, S-U show spores treated with 0.3% Duty Cycle. Arrows indicate areas of spore damage.

Table S1. Microwave pulsed duty cycles used in this study. The varying percentage duty cycles used when microwaving *C. difficile* spores is listed. The duty cycles range from 100% (continuous microwave power) to 0.3% pulsed microwaves. The time microwave power is on and off is shown in milliseconds. Spores were microwaved for 5 seconds in total, which is related to the total number of pulsed microwaves (N) in the table.

Microwaving Duty Cycle (%)	Time Microwave power On (milliseconds)	Time Microwave power Off (milliseconds)	Number of Pulsed microwaves (N)
**100%**	500	0	1 continuous
**33%**	330	670	5
**10%**	100	900	5
**3%**	30	970	5
**1%**	10	990	5
**0.3%**	3	999	5

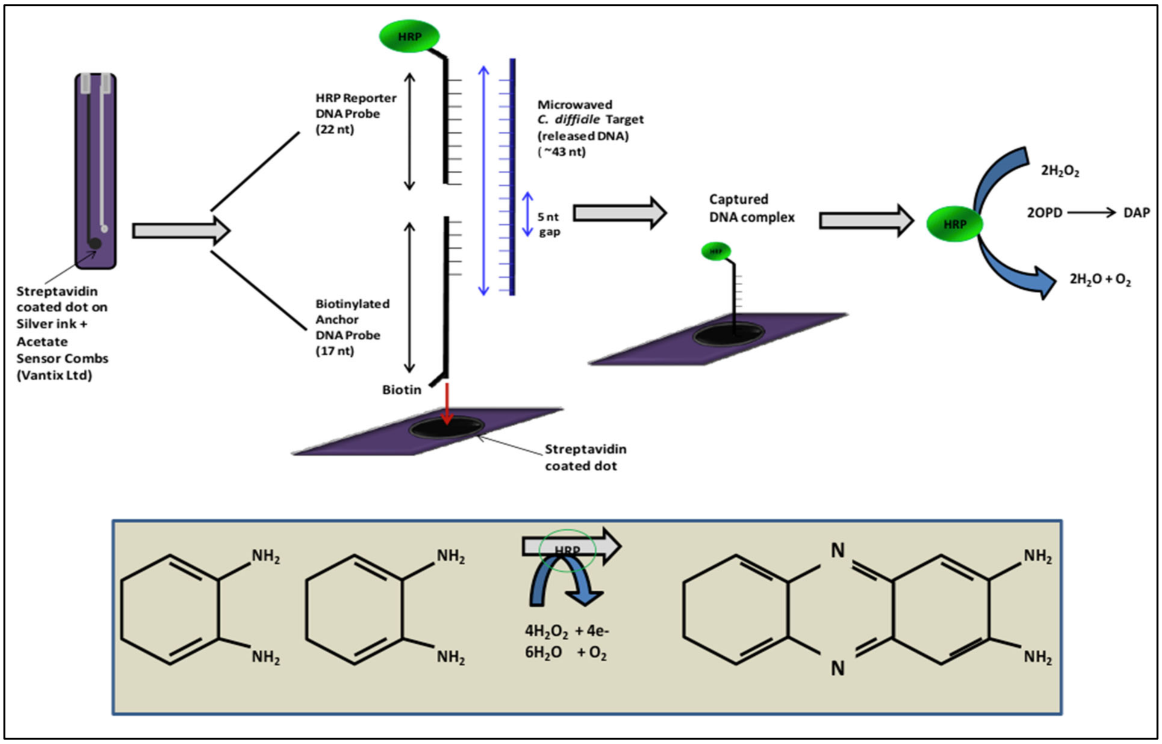

Figure S2. Schematic demonstrating the three piece DNA assay and the chemical detection of HRP. This DNA assay was used to detect both toxin A and toxin B detection. The anchor probe is 17 nucleotides in length and anchored to the streptavidin sensor via addition of a biotin label. The reporter probe (22 nucleotides in length) was attached to an HRP at the 3′ end. Once hybridisation and washing has occurred, the DNA sandwich complex is formed and the HRP can be detected.
